# FNC efficiently inhibits mantle cell lymphoma growth

**DOI:** 10.1371/journal.pone.0174112

**Published:** 2017-03-23

**Authors:** Yan Zhang, Rong Zhang, Xixi Ding, Bangan Peng, Ning Wang, Fang Ma, Youmei Peng, Qingduan Wang, Junbiao Chang

**Affiliations:** 1 Institute of Medical and Pharmaceutical Sciences, Zhengzhou University, Henan, China; 2 Department of Endoscopy, Sun Yat-sen University Cancer Center, State Key Laboratory of Oncology in South China, Collaborative Innovation Center for Cancer Medicine, Guangzhou, China; 3 School of Pharmaceutical Sciences, Zhengzhou University, Zhengzhou, China; 4 College of Chemistry and Molecular Engineering, Zhengzhou University, Zhengzhou, China; University of Kansas Medical Center, UNITED STATES

## Abstract

FNC, 2'-deoxy-2'-β-fluoro-4'-azidocytidine, is a novel cytidine analogue, that has shown strong antiproliferative activity in human lymphoma, lung adenocarcinoma and acute myeloid leukemia. In this study, we investigated the effects of FNC on mantle cell lymphoma (MCL) and the underlying mechanisms. In in vitro experiments, cell viability was detected by the CCK8 assay, and cell cycle progression and apoptosis were assessed by flow cytometry, and the expression of relative apoptosis proteins were detected by Western Blot. The in vivo antitumor effect of FNC was investigated in a SCID xenograft model. Finally, the mechanisms of action of FNC were assessed using a whole human genome expression profile chip. The data showed that FNC inhibited cell growth in a dose- and time-dependent manner, and FNC could induce apoptosis by the death recepter pathways in JeKo-1 cells and arrest the cell cycle in the G1/S or G2/M phase. Notably, FNC showed in vivo efficacy in mice bearing JeKo-1 xenograft tumors. Gene expression profile analysis revealed that the differentially expressed genes were mainly focused on the immune system process, cellular process and death. These findings implied that FNC may be a valuable therapeutic in mantle cell lymphoma and provided an experimental basis for the early clinical application of FNC.

## Introduction

Mantle cell lymphoma (MCL), a B-cell neoplasm, constitutes 6% of the total non-Hodgkin lymphoma (NHL) population[1]. Presently, MCL is a very dangerous disease clinically because it possesses characteristics of both indolent and aggressive lymphomas, and has a more aggressive disease course[2]. The genetic hallmark of MCL is the chromosomal translocation t(11; 14) (q13; q32), which leads to the overexpression of the cell cycle regulatory protein cyclin D1[3]. Despite this common genetic lesion, the clinical behavior and biology of mantle cell lymphoma are highly variable[4]. MCL is responsive to various initial therapies, but relatively short-term remissions are achieved with conventional chemotherapy regimens. Virtually all patients are destined to relapse, and it remains an incurable disease with a rather short median survival of 3–5 years[5–7]. Both high-dose chemotherapy and stem cell transplant cannot change its natural processes. Currently, there is no curative therapy available for refractory MCL[8]. Although using combination regimens has proven to be effective clinically, the development of chemoresistance and side effects in tumor cells is the main obstacle to treatment success. Therefore, there is an urgent need to develop new and more effective anticancer medications for MCL.

Cytotoxic nucleoside analogs were among the first chemotherapeutic agents used in cancer treatment[9]. Nucleoside analogs, including purine and pyrimidine nucleoside derivatives, such as cytarabine, gemcitabine and fludarabine, have been widely used for the treatment of tumors and malignant blood diseases[10–12]. Because of similar chemical construction to normal metabolic nucleotides, this family of compounds behaves as antimetabolites, compete with the cellular endogenous deoxynucleotides and interact with many intracellular targets to induce cytotoxicity[13]. Nucleoside drugs can exert cytotoxic activity by incorporation into and altering the DNA and RNA macromolecules themselves, eventually modifying the metabolism of physiological nucleosides[14]. Better understanding of the molecular mechanisms of the anticancer nucleoside activity may uncover more therapeutic strategies and improve their antitumor efficacy.

FNC, 2'-deoxy-2'-beta-fluoro-4'-azidocytidine, is a novel pyrimidine analog. Presently, a patent application has been submitted for FNC as an invention (patent number: ZL201010506595.X). Previous research has demonstrated that FNC has a significant inhibitory effect on the proliferation of several non-Hodgkin's lymphoma (NHL) cell lines. Similar effects were also seen in various human cancer cell lines, such as lung adenocarcinoma and acute myeloid leukemia[15]. Furthermore, FNC could remarkably inhibit the adhesion, migration and invasion of Raji and JeKo-1 cell lines[16]. Based on our previous work, the mantle cell lymphoma cell line JeKo-1 was chosen to further study the effects of FNC on the proliferation of MCL and its molecular mechanism, providing an experimental basis for the early clinical application of FNC.

## Materials and methods

### Cells and reagents

The MCL JeKo-1 cell line was purchased from the Cell Bank of the Chinese Academy of Sciences (Shanghai, China). The cells were grown in RPMI-1640 medium supplemented with 20% fetal bovine serum and penicillin (100 U/ml)/streptomycin (100μg/ml) at 37°C with 5% CO_2_. The FNC was provided by Professor Junbiao Chang (Zhengzhou University, Zhengzhou, China), and the cytarabine hydrochloride for injection was commercially available (Ara-C; Sinopharm A-Think Pharmaceuticals co., Ltd, China). For in vitro experiments, FNC and Ara-C were dissolved in sterile phosphate-buffered saline (PBS) as a stock solution and then were diluted with culture medium to the desired concentration. For in vivo experiments, FNC and Ara-C were dissolved in 0.9% sodium chloride.

### Cell proliferation analysis

Cell viability was determined using the Cell Counting kit-8 (Dojindo Company, Japan)[17]. Briefly, 2×10^4^ JeKo-1 cells were incubated in a 96-well plate in the absence or presence of various concentrations of FNC and Ara-C. After treatment for the indicated times (24, 48, and 72 h), 20 μL of CCK-8 reagent was added to each well, and the plate was incubated at 37°C for 3 h. The number of cells was measured with a microplate reader at a test wavelength of 450 nm. Each experiment was performed in triplicate and was repeated at least three times.

### Cell cycle analysis

The cell cycle distribution was analyzed by flow cytometry using a cell cycle kit purchased from Br-green Biotechnology (Hubei, China). Exponentially growing JeKo-1 cells (5×10^5^ cells/ml) were treated with different concentrations of FNC (0.0–1.0 μM) for 48 h and then were collected and washed with PBS buffer, fixed in ice-cold ethanol (100%) to a final concentration of 70% and incubated at 4°C overnight. The fixed cells were washed with PBS and incubated with 100 μl of RNase A for 30 min at 37°C, and then 400 μl of propidium iodide (PI) solution was added to the suspension. After staining with PI for 30 min in the dark at room temperature, the samples were analyzed using a Becton Dickson flow cytometer, and the cell cycle profile was analyzed by ModFit LT software (Verity Software House, Topsham, ME, USA). Each assay was repeated three times.

### Apoptosis analysis

The Annexin V-FITC apoptosis detection kit (Keygenbio, Nanjing, China) was used to detect the fraction of apoptotic JeKo-1 cells. Briefly, 5×10^5^ cells per well were exposed to various concentrations of FNC (0, 0.0625, 0.125, 0.25 and 1μM) and were incubated for 48 h. The treated cells were harvested and rinsed with PBS, and then were resuspended in 500 μL of Binding Buffer, followed by the addition of 5 μl of Annexin V-FITC and 5 μL of propidium iodide. After incubation for 15 min at room temperature in the dark, the samples were analyzed by flow cytometry (BD FACS Calibur). Each assay was repeated three times.

### Western blot analysis

Jeko-1 cells were lysed with RIPA cell lysis buffer that contained 1% protease inhibitor cocktail. Total proteins were extracted and separated by 8%-15% SDS-PAGE, and transferred on a poly vinylidene fluoride (PVDF) membrane. The membranes were blocked in 5% skim milk for 1h at room temperature, and then probed with the appropriate primary antibodies at 4°C overnight. After washing in TBST (3×10 min), membranes were incubated with horseradish-peroxidase-conjugated secondary antibody at 37°C for 2h. Then the membrane preparations were washed three times as above. Protein bands were detected with the ECL chemiluminescence Kit. Blots were stripped and photographed and the signal intensity of each band was determined using Image J software.

### In vivo tumor xenograft model

SCID mice inoculated with JeKo-1 cells were used for the evaluation of the function FNC in MCL. Our study was approved by the Animal Ethics Committee of Zhengzhou University. All animals were treated according to the procedures outlined in the Guide for the Care and Use of Laboratory Animals (P. R. China), and all efforts were made to minimize suffering to animals Fifty male SPF-SCID/Beige mice (5–6 weeks old; weighing 18–20 g) (Vital River Laboratory, Beijing, China), were maintained in a specific pathogen free (SPF) environment with a 12 h light/dark cycle at 20−25°C with a relative humidity of 40−70% and received sterilized food and water freely available. The mice were irradiated by X-ray before the inoculation experiment. Next, 200 μL of the tumor cell suspension containing 2×10^7^ cells was injected subcutaneously into the right subaxillary region of SCID mice. Fifteen days later, the average tumor volume reached 200 mm^3^. The tumor-bearing mice were then randomly divided into five groups, and each group had ten mice: negative control group (0.9% sodium chloride injection), positive control group (cytarabine, 36 mg/kg), and low-dose (1 mg/kg), medium-dose (2 mg/kg) and high–dose (3 mg/kg) FNC groups. After treatment started, the general condition, change in tumor growth and weight of the SCID mice were observed every other day, and tumor size was measured using a Vernier caliper. Tumor volumes were calculated as described, V (mm^3^) = length (mm) × (width (mm))^2^ × 0.5. The mice were injected with drugs via the caudal vein at the same time every day. All injections were performed using sterile technique with efforts made to minimize trauma to the animals. After 8 days of treatment, the mice were weighed and euthanized using CO2 inhalation. The tumors were harvested and weighed, and the tumor growth inhibitory rate was calculated. The tumor inhibition rate (TIR%) in the treated versus control mice was expressed as follows: TIR% = (mean tumor weight of the control group—mean tumor weight of the treated group)/(mean tumor weight of the control group)×100%. All of the tumor tissue samples were immediately snap-frozen in liquid nitrogen. The liver and kidney of animals were collected and fixed in 4% buffered paraformaldehyde and paraffin-embedded for hematoxylin and eosin (HE) staining.

### Gene expression profiling

Total RNA extracted from xenograft tumor tissues of the 3 mg/kg FNC and negative control groups were subjected to gene expression profile analysis. Each group has three biological repetitions. The microarray assay was conducted by Shanghai Bio Corporation (Shanghai, China). The expression profiling of all of the samples was tested using the Agilent Whole Human Genome Oligo Microarray (4×44K; Agilent Technologies, Palo Alto, CA), which represents >41,000 human genes and transcripts. After applying the SAS platform online analysis system to analyze the chip results, genes with more than 2-fold changes in the expression level were selected for further analysis.

### Semi-quantitative RT-PCR

Semi-quantitative determination of E2F1,CDKN1A,MKI67,CCND1 and Raf1 gene expression was assessed and compared with the results of the gene expression profile chip. Total RNA was extracted from the tumor tissues of 3 mg/kg FNC and negative control groups using Trizol (ComWin, Beijing, China). Next, 1 μg of RNA was used to synthesize cDNA using ReverTra Ace (Toyobo, Japan) according to the manufacturer’s protocol. The primers used for RT-PCR are presented in Table 1. Human GAPDH was used as an internal control to normalize the mRNA levels. PCR amplification was carried out in a 50-μl reaction mixture with 25 μl of 2×Taq Master Mix (ComWin, Beijing, China), 19 μl of ddH2O, 2 μl (10μM) each of forward and reverse primers and 2 μl of cDNA. The PCR conditions included an initial denaturation at 94°C for 2 min followed by 40 amplification cycles consisting of denaturation for 30 sec at 94°C, annealing for 30 sec at 60°C and extension for 30 sec at 72°C, followed by a final extension for 2 min at 72°C. The annealing temperature was 60°C for GAPDH. PCR products were fractionated on 2% agarose gels and were photographed with a UV gel imaging system (Kodak, USA) using ImageJ software to analyze the gray values. Each assay was repeated three times.

**Table 1:** RT-PCR primers sequences and fragment.

Symbol	Sequence (5′→3′)	Product size (bp)
GAPDH	forward: 5’-CAGGAGGCATTGCTGATGAT-3’	138
	reverse: 5’-GAAGGCTGGGGCTCATTT-3’	
CDKN1A	forward:5′-CCTGGCACCTCACCTGCTCTGCTG-3′	280
	reverse: 5′-GCAGAAGATGTAGAGCGGGCCTTT-3′	
E2F1	forward: 5′-CATCCAGCTCATTGCCAAGAAG-3′	391
	reverse:5′-GATCCCACCTACGGTCTCCTCA-3′	
MKI67	forward:5′-ATTGAACCTGCGGAAGAGCTGA-3′	105
	reverse: 5′-GGAGCGCAGGGATATTCCCTTA-3′	
CCND1	forward:5'-AGGAACAGAAGTGCGAGGAGG-3′	192
	reverse: 5'-GGATGGAGTTGTCGGTGTAGATG-3′	
Raf1	forward: 5'-TCTACACCTCACGCCTTCACC-3′	142
	reverse: 5'-CATCCTCAATCATCCTGCTGTCC-3′	

### Statistical analysis

The IBM SPSS 21.0 software was used. The results were presented as the means±SD. Differences between groups were evaluated by t-test. A P value < 0.05 was considered to be statistically significant. In the gene chip analysis, differentially expressed genes were mainly subjected to fold change analysis, hierarchical clustering analysis, principal component analysis, GO enrichment analysis and pathway enrichment analysis.

## Results

### FNC inhibits the growth of JeKo-1 cells in a dose- and time-dependent manner

**Fig 1:**
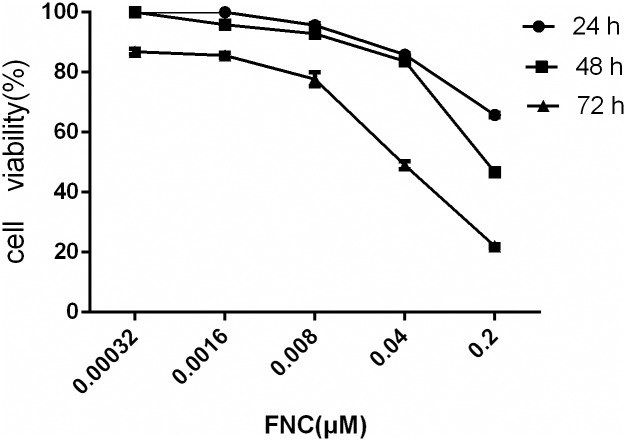
Anti-proliferative activity of FNC on JeKo-1 cells. Detected by CCK-8 assay after 24, 48 and 72 h treatment. Values are Means±SD (means of three independent experiments). FNC shows a dose-and time-dependent anti-proliferative activity in JeKo-1 cells.

The inhibitory effect on cell growth of JeKo-1 was measured by the CCK-8 assay in five treated or untreated groups (0.00032,0.0016,0.008,0.04,0.2 μM and control) for three different treatment times (24 h, 48 h and 72 h), and cytarabine was used as a positive control drug. Here, we showed that FNC significantly inhibited the growth of JeKo-1 cells in a dose- and time-dependent manner (Fig 1). The IC_50_ values of FNC treatment were 0.29 μM,0.18 μM,0.097 μM at 24 h,48 h and 72 h, respectively. We also found that cytarabine inhibited the proliferation of JeKo-1 cells in a dose- and time-dependent manner. The IC_50_ values of cytarabine treatment were 0.48 μM,0.30 μM,0.11 μM at 24 h,48 h and 72 h, respectively.

### FNC induces cell cycle arrest in the G1/S or G2/M phase

**Fig 2:**
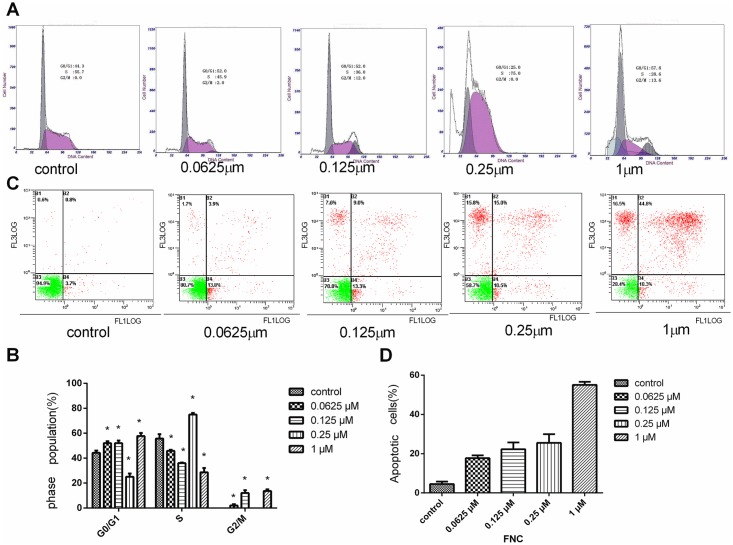
Effects of FNC on cell cycle distribution and apoptosis. (A)FNC induces G1/S or G2/M phase cell cycle arrest. JeKo-1 cells were incubated with 0, 0.0625, 0.125, 0.25, 1 μmol/L FNC for 48 h. The cell cycle distribution was determined via flow cytometry. Data are representative of one of three similar experiments. (C)JeKo-1 cells were treated with 0, 0.0625, 0.125, 0.25, 1 μmol/L FNC for 48 h and harvested. Flow cytometry was performed to observe apoptosis rates. Data are representative of one of three similar experiments. (B and D) Quantified histograms display the effect of FNC on JeKo-1 cells cycle distribution and apoptosis. Data are expressed as means ± SD for 3 independent experiments. * P<0.05 versus the control.

The possible effect of FNC on cell cycle progression in JeKo-1 cells was assessed by flow cytometry. The cells were treated with FNC (0, 0.0625, 0.125, 0.25,and 1 μM) for 48 h. The cell cycle distribution was not the same in different concentrations of FNC (Fig 2A and 2B). FNC (0.0625,0.125,1 μM) treatment significantly increased the proportion of JeKo-1 cells in the G0/G1 phase and G2/M phase compared with the control cells(52.0±1.51%,52.0±2.12% and 57.8±2.40% vs. 44.3±1.78%, p<0.05; 2.0±1.02%,12.0±2.33% and 13.6±1.35% vs. 0.0, p<0.05). At a concentration of 0.25 μmol/L, FNC induced arrest in the S phase in JeKo-1 cells (75.0±1.36% vs. 55.7±3.55%, p<0.05). Taken together, our results showed that FNC could cause cell cycle arrest at any one phase, and FNC is a cell cycle-nonspecific agent (CCNSA).

### FNC induces the apoptosis of JeKo-1 cells

To further investigate whether the growth inhibition was due to apoptosis, JeKo-1 cells were incubated with FNC (0, 0.0625, 0.125, 0.25 and 1 μM) for 48 h and then were analyzed by flow cytometry. As shown in Fig 2C and 2D, the percentages of apoptotic cells were significantly increased by FNC treatment in a dose-dependent manner (P<0.05). The percentage of cells undergoing apoptosis was determined by the sum of the cells in early and late apoptosis.

**Fig 3:**
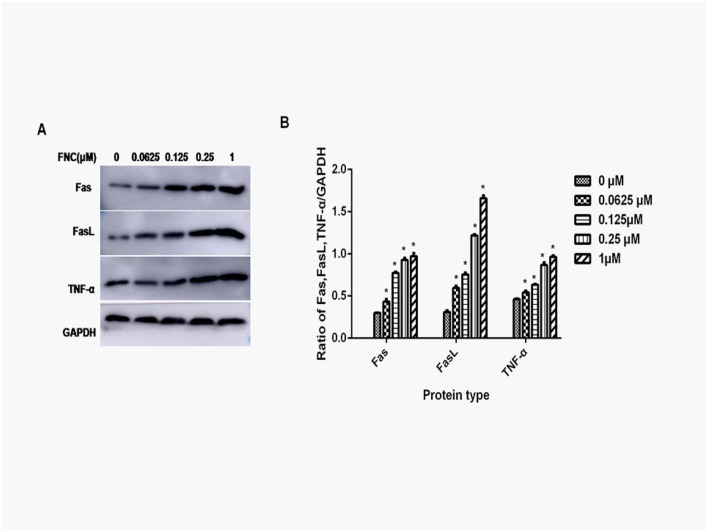
Effect of FNC treatment on Fas, FasL, TNF-α protein expression in Jeko-1 cells. (A) Western blot of proteins extracted from Jeko-1 cells following 48h treatment with FNC (0μM, 0.0625μM, 0.125μM, 0.25μM and 1.000μM). A representative result of 3 independent experiments is shown. (B) The Fas/GAPDH, FasL/GAPDH and TNF-α/GAPDH ratio is displayed as Mean±SD. * P<0.05 versus the control.

Jeko-1 cells were treated with FNC (0, 0.0625, 0.125, 0.25 and1 μM) for 48 h followed by Western blotting analysis of protein expression. Following FNC treatment, Fas, FasL and TNF-α expression significant increased (Fig 3).

### FNC inhibits tumor growth in SCID mice

**Fig 4:**
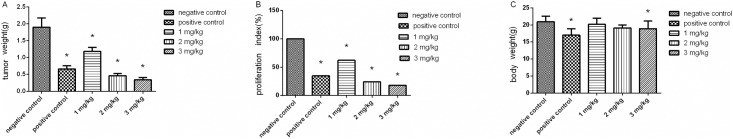
FNC significantly inhibited the growth of human JeKo-1 xenografts in vivo. (A)Graphs represent the average weight of tumors from every group. (B)shows the average proliferation index(proliferation index% = (tumor weight of drug group/tumor weight of negative control group)×100%]. (C) Graphs represent the average body weight, the body weights were measured after treatment. Tumor weights and body weights are presented as means±SD. * P<0.05 versus the control.

**Fig 5:**
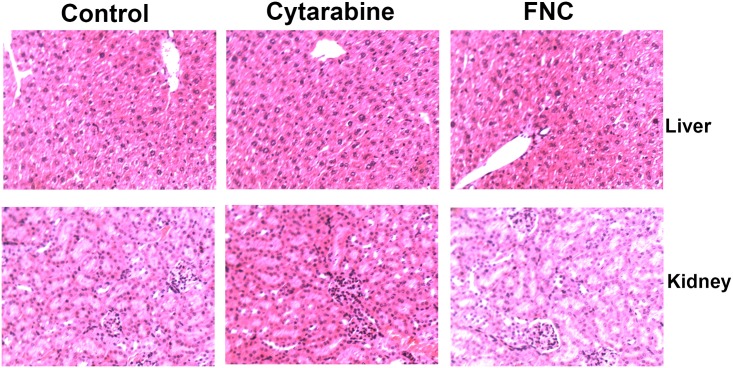
Histopathological examination of liver and kidney in FNC treated mice. When the mice were euthanized, liver and kidney were collected and fixed in 4% buffered paraformaldehyde and paraffin embedded for H&E staining. Pictures were original captured at 100× magnification.

To evaluate the antitumor activity of FNC in vivo, JeKo-1-bearing SCID/Beige mice were injected with FNC via the caudal vein. When administered at a dose of 1, 2 or 3 mg/kg for eight consecutive days, the inhibitory rates were 37.9%,75.8% and 82.1%, respectively. At the end of the experiment, tumor volume was 1857.73 ± 326.51 mm^3^ in the control group, while tumor volume in the treatment groups was 1089.35± 267.14 mm^3^, 452.65 ± 96.38 mm^3^ and 274.40± 77.26 mm^3^ at FNC doses of 1 mg/kg, 2 mg/kg and 3 mg/kg, respectively. The tumor volume in cytarabine group was 553.61 ± 123.85 mm^3^. Our results showed that the tumor growth in the FNC-treated group was markedly inhibited compared with that of the negative control group, and FNC significantly suppressed tumor growth in a dose-dependent manner (Fig 4A and 4B). Furthermore, the inhibitory rate of cytarabine (36 mg/kg) was 65%. In addition, the body weight of mice had significantly decreased at a dose of 3 mg/kg FNC and 36 mg/kg cytarabine (Fig 4C). In the positive control group, two mice died during the treatment. Otherwise, the low- and medium-dose FNC groups did not cause significant body weight loss compared with the negative control group. Histopathological examination of the liver and kidney revealed no signs of toxicity to the organ tissues (Fig 5).

### Microarray analysis of gene expression in xenograft tissue

**Fig 6:**
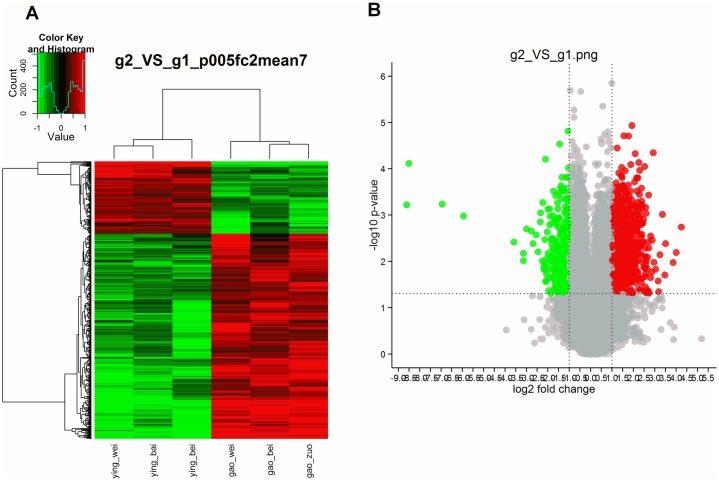
Gene expression profiling analysis of differential expression genes in xenograft tissue with and without FNC treatment. Red color represents the up-regulated genes, and green color represents the down-regulated genes. (A)Hierarchical clustering(p≤0.05, fc≥2 or fc≤0.5, mean = 7). (B)Volcano plot. The x axis represents the differential expression profiles with the fold-induction ratios in a log2 scale, and the y axis represents the P value of T-test in a log10 scale. Differentially expressed genes are established at Fold change ≥2 and P < 0.05.

**Fig 7:**
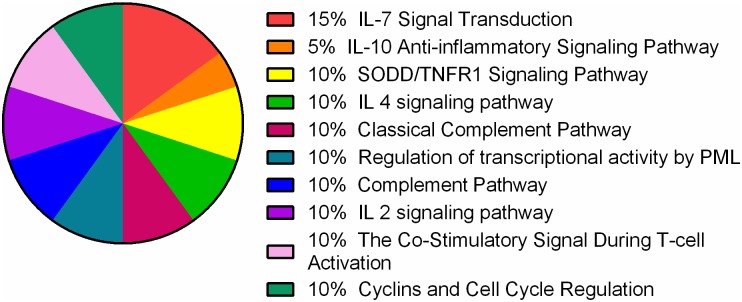
Pie chart of pathway enrichment. We screen out the top 10 significant enrichment pathway terms based on Biocarta. The percentage of each pathway is shown.

To obtain more comprehensive insight into the mechanisms by which FNC exerts its anti-tumor effects, we carried out a microarray study of xenograft tissue following exposure to FNC[18;19]. The gene expression profiles of the medicine group (3 mg/kg FNC) were compared with that of the negative control group using the Agilent Whole Human Genome Oligo Microarray (4×44K). The report of total RNA sample quality certification are showed in S1 Table and S1 Fig. The sample we use is uniform and comparable(S2 Fig). Our experimental design is reasonable and the biology repeatability is good(S3 Fig). The genes were filtered by the setting criterion of p<0.05,fold change≥2 (up-regulated) or≤0.5 (down-regulated) and mean = 7 (significant differences are reflected in the three biological repetitions) as significantly differentially expressed genes. First, we found that there were 606 genes that were significantly different between the 3.0mg/kg FNC and vehicle control group. As shown in Fig 6A and 6B, 448 genes were up-regulated (74%), and 158 genes were down-regulated (26%). S2 and S3 Tables shows the most significant difference gene in up-regulated and down-regulated genes respectively. By pathway enrichment analysis(http://cgap.nci.nih.gov/Pathways/BioCarta Pathways), we found that the differentially expressed genes were mainly involved in cell cycle and immunity based on the Biocarta database. Fig 7 shows the top 10 significantly enriched pathway terms. Many factors, including IL2RG,JAK3,IL7R,TNF,BIRC3,PML and CCND2, were involved in those pathways(S4 Table). GO analysis was also performed(http://geneontology.org/), and the enriched GO-terms (Enrichment test p value = 0) in the ontology classification “Biological Process” were selected and are presented in Table 2. The results showed that “cellular process” contained the greatest number of genes among the GO-terms; thus, we analyzed the main GO terms of cellular process (Enrichment test p value≤0.001) and present them in Table 3. Furthermore, we identified genes related to cell proliferation, cell cycle and apoptosis, respectively, as shown in Tables 4, 5, 6 and 7.

**Table 2:** Table analysis of GO enrichment.

GOID	Name	P	Hits
GO:0002376	immune system process	0	94
GO:0009987	cellular process	0	352
GO:0016265	death	0	65
GO:0032501	multicellular organismal process	0	159
GO:0032502	developmental process	0	145
GO:0050896	response to stimulus	0	163
GO:0051704	multi-organism process	0	44
GO:0065007	biological regulation	0	250
GO:0048518	positive regulation of biological process	0	111
GO:0048519	negative regulation of biological process	0	94
GO:0050789	regulation of biological process	0	229

Hits: Number of genes included in the go term.

**Table 3:** The main GO terms of cellular process.

GOID	Name	P	Hits
GO:0001775	cell activation	0	39
GO:0007154	cell communication	0	148
GO:0007155	cell adhesion	7.00E-04	38
GO:0008219	cell death	0	65
GO:0008283	cell proliferation	0	55
GO:0048522	positive regulation of cellular process	0	97
GO:0048523	negative regulation of cellular process	3.00E-04	79
GO:0050794	regulation of cellular process	0	216

**Table 4:** Positive regulation of cell proliferation.

InputId	GeneId	foldchange	Symbol	InputId	GeneId	foldchange	Symbol
A_23_P109988	942	2.01	CD86	A_23_P55099	5578	2.80	PRKCA
A_23_P137196	3597	3.31	IL13RA1	A_23_P57364	7032	2.52	TFF2
A_23_P138760	23529	2.36	CLCF1	A_23_P59210	1026	2.45	CDKN1A
A_23_P14774	1512	2.02	CTSH	A_23_P86390	8829	2.20	NRP1
A_23_P167328	952	0.32	CD38	A_24_P173823	5087	2.13	PBX1
A_23_P17998	3280	2.27	HES1	A_24_P245298	8742	2.40	TNFSF12
A_23_P208493	10288	2.10	LILRB2	A_24_P302584	6664	0.48	SOX11
A_23_P28857	55423	3.97	SIRPG	A_24_P407645	6693	0.44	SPN
A_23_P327380	8626	2.78	TP63	A_24_P938293	3280	2.25	HES1
A_23_P372874	6284	2.03	S100A13	A_32_P65616	5617	3.84	PRL
A_23_P52017	259266	0.44	ASPM				

**Table 5:** Negative regulation of cell proliferation.

InputId	GeneId	foldchange	Symbol	InputId	GeneId	foldchange	Symbol
A_23_P109143	5621	2.04	PRNP	A_23_P59210	1026	2.45	CDKN1A
A_23_P164650	348	2.40	APOE	A_23_P76364	928	0.46	CD9
A_23_P168882	94241	4.27	TP53INP1	A_24_P103469	7940	4.35	LST1
A_23_P1962	5920	2.40	RARRES3	A_24_P12651	23087	2.31	TRIM35
A_23_P208493	10288	2.10	LILRB2	A_24_P207139	5371	2.18	PML
A_23_P211207	104	0.27	ADARB1	A_24_P228130	414062	0.29	CCL3L3
A_23_P259490	10608	3.06	MXD4	A_24_P234732	10608	2.92	MXD4
A_23_P28857	55423	3.97	SIRPG	A_24_P302584	6664	0.48	SOX11
A_23_P321920	414062	0.49	CCL3L3	A_24_P379104	11040	2.10	PIM2
A_23_P376488	7124	3.99	TNF	A_24_P407645	6693	0.44	SPN
A_23_P423695	10608	3.47	MXD4	A_24_P94916	7940	4.75	LST1
A_23_P55099	5578	2.80	PRKCA				

**Table 6:** Cell cycle arrest related genes covered in GO.

InputId	GeneId	foldchange	Symbol
A_23_P109143	5621	2.04	PRNP
A_23_P168882	94241	4.27	TP53INP1
A_23_P314760	51422	2.02	PRKAG2
A_23_P35082	83667	2.58	SESN2
A_23_P361448	143686	2.37	SESN3
A_23_P436259	2081	2.45	ERN1
A_23_P59210	1026	2.45	CDKN1A
A_24_P207139	5371	2.18	PML
A_24_P921823	6934	0.40	TCF7L2

**Table 7:** Genes related to apoptosis.

GoId	Name	InputId	Geneid	foldchange	Symbol
GO:0045768	positive regulation of anti-apoptosis	A_23_P59210	1026	2.45	CDKN1A
GO:0060561	apoptosis involved in morphogenesis	A_24_P20719	5371	2.18	PML

### Verification of the microarray data by RT-PCR

**Fig 8:**
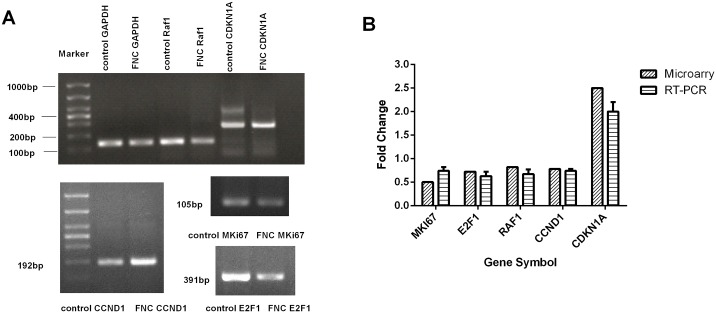
Verification of microarray data by RT-PCR. (A) Electrophoresis image of agarose gel. GAPDH was used as an internal control. The pictures shown are representatives of 3 independent experiments. (B)Gene expression levels of 5 selected genes. The right panel of each gene shows the gene expression levels as detected by RT-PCR, and the left panel of each gene shows the gene array data. It shows that the result of RT-PCR was consistent with the microarray results.

Selected genes with altered expression levels by microarray analysis were confirmed by RT-PCR. The mRNA expression of E2F1,MKI67,CCND1 and Raf1 detected by RT-PCR were down-regulated, and the mRNA expression of CDKN1A was up-regulated (Fig 8). The results showed acceptable consistency between the results of RT-PCR and microarray data.

## Discussion

MCL is considered one of the most aggressive lymphomas with poor responses to conventional chemotherapy and a relatively short survival[20]. Therefore, novel, more effective and less toxic anti-carcinogens are needed to improve the curative effect on MCL. In the current study, we first demonstrated that FNC efficiently inhibited the proliferation of Jeko-1 cells both in vitro and in vivo and investigated the possible mechanism. In vitro, FNC markedly inhibited the growth of JeKo-1 cells in a dose- and time-dependent manner. Moreover, compared with the positive control drug (cytarabine), the dose of FNC was smaller. In vivo, we used the human-derived MCL cell line JeKo-1 with SCID mice to establish a xenograft model, and the tumor formation rate was 100%. The in vivo experiment further revealed that FNC significantly suppressed tumor growth in a dose-dependent manner. This finding was of particular importance because it was the first time that FNC was shown to inhibit JeKo-1 tumor growth in vivo. Both the inhibitory rates of FNC in the medium- and high-dose groups were higher than those of the positive control group. Of importance, cytarabine treatment caused the most significant body weight loss, and two mice died during the cytarabine treatment, while FNC was well-tolerated in xenograft mice. These results suggested that FNC might be a potential anti-MCL candidate for clinical applications.

Recently, remarkable advances have been made in the anti-MCL mechanisms of drugs[21]. Among them, inhibiting proliferation, inducing apoptosis and cell cycle arrest are the main anti-tumor mechanisms[22]. Because defects in apoptosis and cell cycle regulation are primary events in MCL, we investigated the effect of FNC treatment on cell cycle and apoptosis. Our results demonstrated that FNC could induce G1/S or G2/M phase cell cycle arrest, and FNC is a cell cycle-nonspecific agent. Furthermore, FNC significantly induced apoptosis in the JeKo-1 cell line. Obviously, regulating the cell cycle and inducing apoptosis may be one of the important antitumor mechanisms of FNC.

Cell cycle is the basic feature of life activities regulated by a variety of proteases. Cyclin-CDK complexes which directly involved in regulating different cell cycle transitions include CDK4(6)/cyclinD, CDK2/cyclinE, CDK2/cyclinA and CDK1/cyclinB. CyclinD binding to CDK4 and CDK6 plays important roles in the G1 phase. CDK2/cyclinE induces the transition of G1 to S phase. After CyclinA binding to CDK2 and CDK1 respectively, cells complete S phase transformation, proceed to M phase. CDK1/cyclinB controls the access and adjustment of the M phase. Our results show that FNC causes cell cycle arrest of different periods concentration-dependently, which may be related to the concentration-dependent regulation of FNC on cell cycle checkpoint activation.

Apoptosis plays an important role in development of tumor. There are two major apoptosis pathways: the cell death receptor-mediated extrinsic pathway and the mitochondrial-mediated intrinsic pathway. According to the results of gene chip, Fas, Fasl and TNF-α were chosen to observe the effect of FNC on Jeko-1 cells. Death receptors are transmembrane proteins belonging to tumor necrosis factor receptor (TNFR) gene superfamily. Fas and TNFRs are the most important among these receptors. Upon ligand binding, these receptors gather and activate adapter proteins, lead to the activation of downstream caspases and finally induce apoptosis. Death ligands include TNF-α, FasL and tumor necrosis factor related apoptosis inducing ligand (TRAIL). FAS is an important death receptor on cell surface which binds its ligand FASL and activates the process of transmitting signals to induce the target cell apoptosis. TNF-α is a kind of cytokines with many biological effects. TNF is triggered by two kinds of TNF receptor (TNFR) on the cell surface. The major signaling pathways include apoptosis mediated by caspase family, transcription factor NF-κB mediated by adapter protein TRAF, and the activation of JNK protein kinase. We found that FNC treatment significantly increased the protein expression of Fas, FasL and TNF-α. These results indicate that FNC induces apoptosis in Jeko-1 cells via the cell death receptor-mediated extrinsic pathway.

In this study, we compared the gene-expression profiles with and without FNC treatment and searched for genes that were markedly upregulated or downregulated by FNC treatment. 448 genes were found to reveal significantly higher expression levels, and 158 genes had significantly lower expression levels in the FNC (3.0 mg/kg) group than in the negative control group. Pathway analysis of the microarray data revealed that the most significant pathways were involved in the immune system and cell cycle. The most significantly enriched terms such as “immune system process”, “cellular process” and “death” were selected by GO enrichment analysis. Therefore, FNC may affect the occurrence and development of tumors through multiple molecular pathways.

According to the results of gene expression profile chip analysis, genes such as CDKN1A, PML, TP53INP1, TNF, SPN and LST1 are associated with cell growth; PRNP, TP53INP1, PRKAG2, SESN2, SESN3, ERN1, CDKN1A, PML and TCF7L2 are involved in cell cycle arrest; and genes such as CDKN1A, PML, BIRC3, CASP10 and TNF play a role in apoptosis. Particularly, two tumorigenesis-related genes, PML and CDKN1A, are related to all three biological processes. Accordingly, we can infer that PML and CDKN1A are critical genes in these pathways.

As is well known, CDKN1A (also known as p21) is a cyclin-dependent kinase inhibitor[23]. P21 together with P53 make up the cell cycle G1 check point, and p21 also plays a regulatory role in S phase DNA damage repair and DNA replication[24]. In addition to its role in cell cycle control, CDKN1A is involved in the regulation of cellular senescence, gene transcription and apoptosis[25]. In addition, previous studies have reported that PML functions as an important tumor suppressor, and PML regulates numerous fundamental processes, such as apoptosis, cellular proliferation and cell cycle regulation[26]. Furthermore, PML plays a role in a significantly enriched pathway-regulation of transcriptional activity by PML, and this pathway can regulate the expression of CDKN1A through the P53 pathway. Thus, one possible anti-tumor mechanism of FNC was found to be associated with the regulation of transcriptional activity by the PML pathway. Apart from that, many of the significantly enriched pathways found in this study were already reported to be involved in the immune system process. This finding suggests that modulating the immune system may be another important anti-tumor mechanism of FNC, and we will investigate it further.

In conclusion, for the first time, we revealed that FNC could inhibit JeKo-1 cell proliferation and tumor growth in vitro and in vivo. Our in vitro experiments suggest that the underlying mechanisms may be, at least in part, because FNC can cause cell cycle arrest in the G1/S or G2/M phase and induces apoptosis. Therefore, detailed elucidation of the pathways and the respective targets of FNC involved in the cell cycle and apoptosis are critical for its potential application in cancer therapy. In the present study, we first compared the gene-expression profiles with and without FNC treatment to provide a comprehensive analysis of its antitumor mechanisms. Within our observation, FNC regulated many biological process involved in tumors, such as the immune system process, cell proliferation, cell cycle and apoptosis. One possible anti-tumor mechanism of FNC was found to be associated with the regulation of transcriptional activity by the PML pathway. This study shows the potential of FNC as a therapeutic agent for mantle cell lymphoma.

## Supporting information

S1 Fig Electrophoresis image. (TIF)

S2 Fig Box blot. The central line represents the median of the data. The box represents an interval that contains 50% of the data. This range is called the IQR(Inter Quantile Range). The upper and lower edges of each box represent the 75th and 25th percentile, respectively. It shows that all data concentrate in the middle area and there is no discrete distribution data. The sample is uniform and comparable.
(TIF)

S3 Fig PCA(Principal Component Analysis). The distance between the points on the image shows the similarity between samples. It is observed that the distance of three biological repetition in negative control group is very close to each other and so is the drug group. It shows that the biology repeatability is good and the experimental design is reasonable.
(TIF)

S1 Table The report of total RNA sample quality certification. (DOC)

S2 Table The most significant difference gene in up-regulated genes. (DOC)

S3 Table The most significant difference gene in down-regulated genes. (DOC)

S4 Table Differentially expressed genes in the main pathways. (DOC)
