# Supplementary figures and images for: FNC efficiently inhibits mantle cell lymphoma growth
Source: PLoS One. 2017 Mar 23;12(3):e0174112. doi: 10.1371/journal.pone.0174112 (PMC5363836; doi:10.1371/journal.pone.0174112)

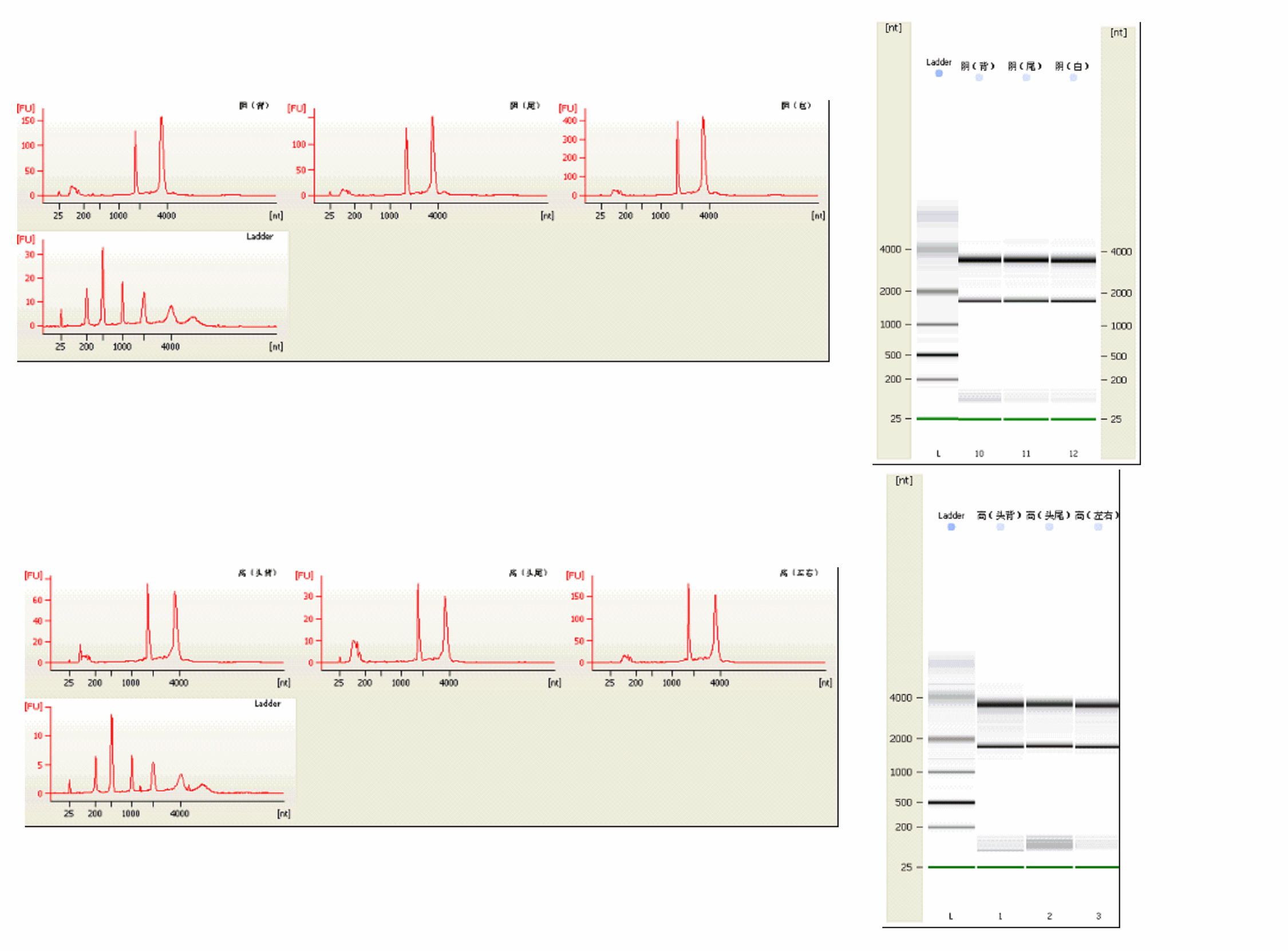

Supplement: S1 Fig — (TIF) [file pone.0174112.s001.tif]

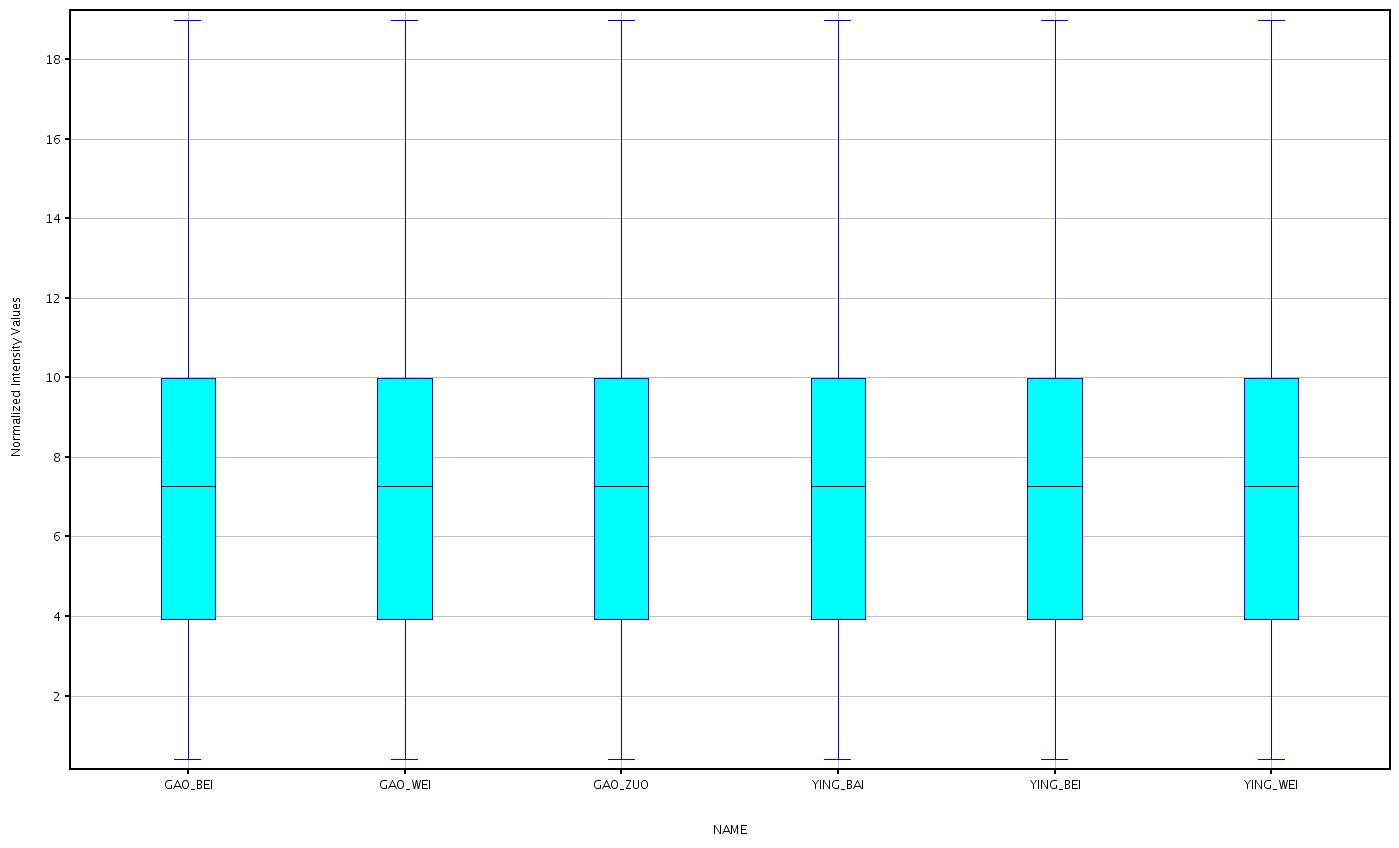

Supplement: S2 Fig — The box represents an interval that contains 50% of the data. This range is called the IQR(Inter Quantile Range). The upper and lower edges of each box represent the 75th and 25th percentile, respectively. It shows that all data concentrate in the middle area and there is no discrete distribution data. The sample is uniform and comparable. (TIF) [file pone.0174112.s002.tif]

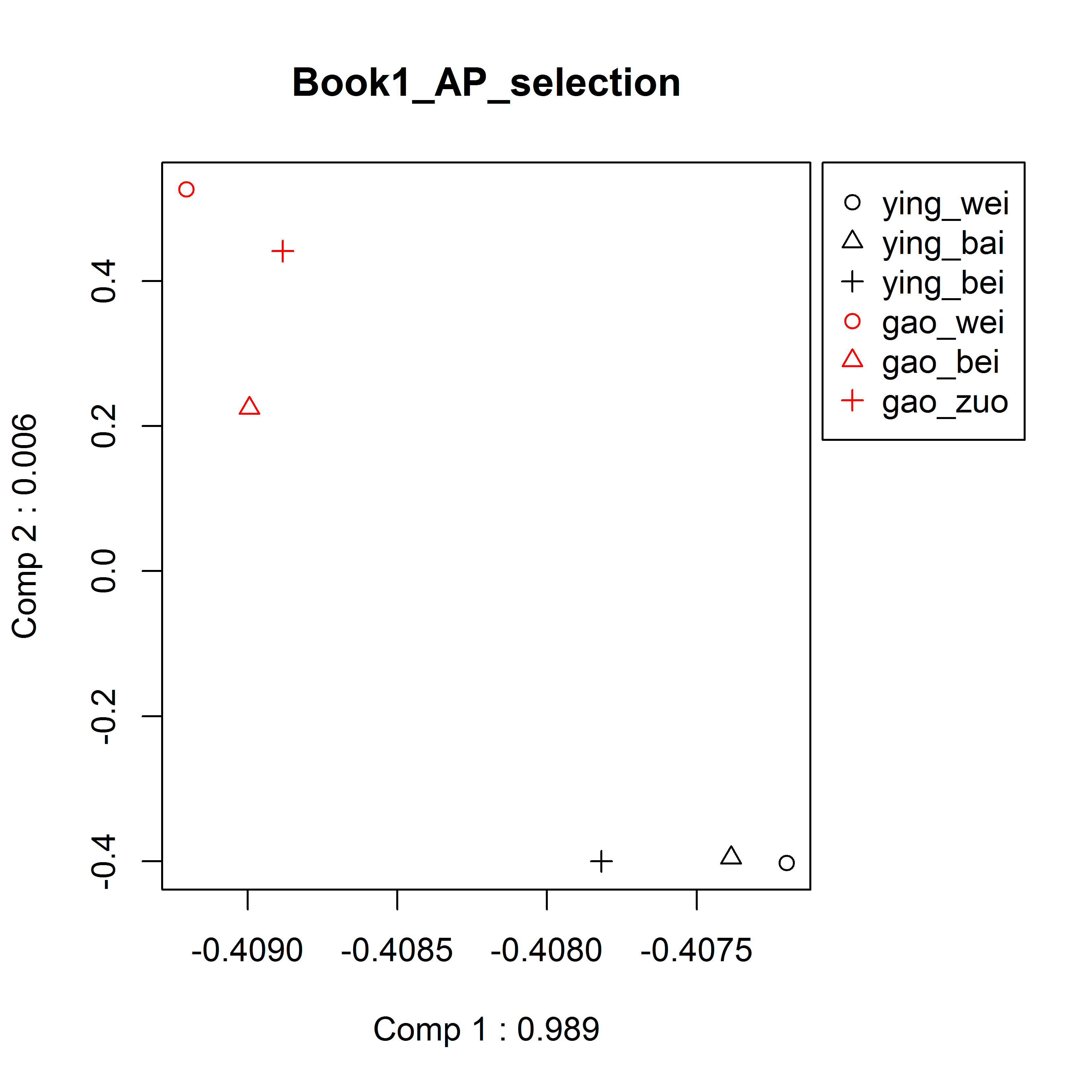

Supplement: S3 Fig — The distance between the points on the image shows the similarity between samples. It is observed that the distance of three biological repetition in negative control group is very close to each other and so is the drug group. It shows that the biology repeatability is good and the experimental design is reasonable. (TIF) [file pone.0174112.s003.tif]
