# Supplementary material for: FNC efficiently inhibits mantle cell lymphoma growth
Source: PLoS One. 2017 Mar 23;12(3):e0174112. doi: 10.1371/journal.pone.0174112 (PMC5363836; doi:10.1371/journal.pone.0174112)
Supplement: S1 Table — (DOC) [file pone.0174112.s004.doc]

**S1 Table** The report of total RNA sample quality certification

| No. | Con(μg/μL) | Vol(μL) | Total(μg) | A260/ A280 | 2100 Result | | Result |
| --- | --- | --- | --- | --- | --- | --- | --- |
|  |  |  |  |  | RIN | 28S/18S |  |
| 1 | 0.466 | 100 | 46.57 | 1.97 | 10.0 | 2.1 | qualified |
| 2 | 1.099 | 100 | 109.94 | 2.03 | 10.0 | 2.0 | qualified |
| 3 | 1.097 | 100 | 109.66 | 2.06 | 9.9 | 2.0 | qualified |
| 4 | 1.300 | 100 | 130.02 | 2.06 | 9.5 | 1.6 | qualified |
| 5 | 0.385 | 100 | 38.53 | 1.97 | 9.3 | 1.5 | qualified |
| 6 | 0.668 | 100 | 66.77 | 2.02 | 9.4 | 1.7 | qualified |
