# Supplementary material for: FNC efficiently inhibits mantle cell lymphoma growth
Source: PLoS One. 2017 Mar 23;12(3):e0174112. doi: 10.1371/journal.pone.0174112 (PMC5363836; doi:10.1371/journal.pone.0174112)
Supplement: S2 Table — (DOC) [file pone.0174112.s005.doc]

**S2 Table** **The most significant difference gene in up-regulated genes**

| GeneSymbol | pvalues | foldchange | EntrezGeneID | GeneName |
| --- | --- | --- | --- | --- |
| ASB13 | 3.54968E-05 | 2.37 | 79754 | ankyrin repeat and SOCS box containing 13 |
| LST1 | 7.29819E-05 | 4.75 | 7940 | leukocyte specific transcript 1 |
| SMKR1 | 8.92633E-05 | 5.75 | 100287482 | small lysine-rich protein 1 |
| TREML2 | 9.27996E-05 | 2.73 | 79865 | triggering receptor expressed on myeloid cells-like 2 |
| JAK3 | 0.000122209 | 2.48 | 3718 | Janus kinase 3 |
| STK17A | 0.000155146 | 2.76 | 9263 | serine/threonine kinase 17a |
| MAP7D2 | 0.000156866 | 4.44 | 256714 | MAP7 domain containing 2 |
| HES1 | 0.000199646 | 2.27 | 3280 | hes family bHLH transcription factor 1 |
| SLFN5 | 0.000222406 | 3.38 | 162394 | schlafen family member 5 |
| ISG20 | 0.000226285 | 2.86 | 3669 | interferon stimulated exonuclease gene 20kDa |
