# Supplementary material for: FNC efficiently inhibits mantle cell lymphoma growth
Source: PLoS One. 2017 Mar 23;12(3):e0174112. doi: 10.1371/journal.pone.0174112 (PMC5363836; doi:10.1371/journal.pone.0174112)
Supplement: S3 Table — (DOC) [file pone.0174112.s006.doc]

**S3 Table The most significant difference gene in down-regulated genes**

| GeneSymbol | pvalues | foldchange | EntrezGeneID | GeneName |
| --- | --- | --- | --- | --- |
| HBA1 | 7.62109E-05 | 0.00 | 3039 | hemoglobin, alpha 1 |
| ELOVL6 | 0.000151982 | 0.40 | 79071 | ELOVL fatty acid elongase 6 |
| SLC23A1 | 0.000152591 | 0.46 | 9963 | solute carrier family 23 (ascorbic acid transporter), member 1 |
| DLEU2 | 0.000153867 | 0.47 | 8847 | deleted in lymphocytic leukemia 2 (non-protein coding) |
| CCL4 | 0.000259383 | 0.38 | 6351 | chemokine (C-C motif) ligand 4 |
| INSIG1 | 0.000260596 | 0.48 | 3638 | insulin induced gene 1 |
| SPN | 0.000263029 | 0.44 | 6693 | sialophorin |
| CAPN15 | 0.000296923 | 0.40 | 6650 | calpain 15 |
| DKFZP547L112 | 0.000380651 | 0.32 | 81787 | uncharacterized protein DKFZp547L112 |
| MMS22L | 0.000409209 | 0.47 | 253714 | MMS22-like, DNA repair protein |
