# Supplementary material for: FNC efficiently inhibits mantle cell lymphoma growth
Source: PLoS One. 2017 Mar 23;12(3):e0174112. doi: 10.1371/journal.pone.0174112 (PMC5363836; doi:10.1371/journal.pone.0174112)
Supplement: S4 Table — (DOC) [file pone.0174112.s007.doc]

**S4 Table Differentially expressed genes in the main pathways**

| Name | Symbol | pValue | InputId |
| --- | --- | --- | --- |
| IL-7 Signal Transduction | IL2RG | 0.005 | A_23_P148473 |
| IL-7 Signal Transduction | JAK3 | 0.005 | A_24_P59667 |
| IL-7 Signal Transduction | IL7R | 0.005 | A_23_P404494 |
| IL-10 Anti-inflammatory Signaling Pathway | TNF | 0.0213 | A_23_P376488 |
| SODD/TNFR1 Signaling Pathway | TNF | 0.0213 | A_23_P376488 |
| SODD/TNFR1 Signaling Pathway | BIRC3 | 0.0213 | A_23_P98350 |
| IL 4 signaling pathway | IL2RG | 0.0294 | A_23_P148473 |
| IL 4 signaling pathway | JAK3 | 0.0294 | A_24_P59667 |
| Classical Complement Pathway | C1S | 0.0385 | A_23_P2492 |
| Classical Complement Pathway | C4B | 0.0385 | A_23_P42282 |
| Regulation of transcriptional activity by PML | PML | 0.0539 | A_24_P207139 |
| Regulation of transcriptional activity by PML | TNF | 0.0539 | A_23_P376488 |
| Complement Pathway | C1S | 0.0651 | A_23_P2492 |
| Complement Pathway | C4B | 0.0651 | A_23_P42282 |
| IL 2 signaling pathway | IL2RG | 0.0833 | A_23_P148473 |
| IL 2 signaling pathway | JAK3 | 0.0833 | A_24_P59667 |
| The Co-Stimulatory Signal During T-cell Activation | CD3D | 0.0833 | A_23_P138985 |
| The Co-Stimulatory Signal During T-cell Activation | CD86 | 0.0833 | A_23_P109988 |
| Cyclins and Cell Cycle Regulation | CCND2 | 0.0961 | A_24_P278747 |
| Cyclins and Cell Cycle Regulation | RBL1 | 0.0961 | A_24_P276102 |
